# Role of Rph3A in brain injury induced by experimental cerebral ischemia‐reperfusion model in rats

**DOI:** 10.1111/cns.13850

**Published:** 2022-04-25

**Authors:** Xianlong Zhu, Haiying Li, Wanchun You, Zhengquan Yu, Zongqi Wang, Haitao Shen, Xiang Li, Hao Yu, Zhong Wang, Gang Chen

**Affiliations:** ^1^ 74566 Department of Neurosurgery & Brain and Nerve Research Laboratory First Affiliated Hospital of Soochow University Suzhou Jiangsu China; ^2^ Institute of Stroke Research Soochow University Suzhou Jiangsu China; ^3^ Department of Neurosurgery The Second People's Hospital of Lianyungang City Lianyungang Jiangsu China; ^4^ Department of Neurosurgery The First People's Hospital of Nantong city Nantong Jiangsu China

**Keywords:** astrocyte, ischemic stroke, neuron, penumbra, Rph3A

## Abstract

**Aim:**

The aim was to study the role of Rph3A in neuronal injury induced by cerebral ischemia‐reperfusion.

**Methods:**

The protein and mRNA levels of Rph3A in penumbra were detected by Western blot. The localization of Rph3A in different cell types in penumbra was detected by immunofluorescence. Apoptosis in the brain was detected by TUNEL staining. We tested neurobehavioral evaluation using rotarod test, adhesive‐removal test, and Morris Water maze test. We examined the expression and localization of Rph3A in cultured neurons and astrocytes in vitro by Western blot and ELISA, respectively.

**Results:**

The mRNA and protein levels of Rph3A had significantly increased in brain penumbra of the rat MCAO/R model. Rph3A was mainly distributed in neurons and astrocytes and was significantly increased by MCAO/R. We downregulated Rph3A and found that it further worsened the cerebral infarct, neuronal death and behavioral, cognitive, and memory impairments in rats after MCAO/R. We also found that ischemia‐reperfusion upregulated the *in vitro* protein level and secretion of Rph3A in astrocytes but led to a decrease in the protein level of Rph3A in neurons.

**Conclusion:**

The increase in Rph3A in the brain penumbra may be an endogenous protective mechanism against ischemia‐reperfusion injury, which is mainly dominated by astrocytes.

## Graphical abstract

Ischemic stroke is one of the main causes of human death and disability. Rabphilin 3A (Rph3A) is involved in cerebral ischemia‐reperfusion injury (CIRI). The increase in Rph3A in the brain penumbra may be an endogenous protective mechanism against CIRI, which is mainly dominated by astrocytes.

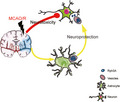

## 1 INTRODUCTION

According to the Global Burden of Disease Study 2017, deaths due to stroke increased by 14.6 per 100,000 people, and stroke is the leading cause of death worldwide.^1^ Given China's aging population, the country faces an even greater challenge related to stroke, which has been increasing for the past 30 years.^2^ According to statistics from Chinese stroke centers, more than 3 million patients were hospitalized for stroke in the past year, of which almost 82% had ischemic strokes. Many patients were discharged with varying degrees of sequelae that seriously affect the quality of life.^3^ Unfortunately, effective treatment strategies for ischemic stroke are limited, and include intravenous thrombolysis and mechanical thrombectomy that aim to restore the blood supply to the affected brain region.^4^ However, there is a potential risk of recanalization, that is, reperfusion injury, manifested by increased cerebral edema, infarct progression, hemorrhagic stroke, and neurological deterioration.^5^ Previous studies have suggested that these changes may be associated with excitotoxicity, neuroinflammation, oxidative stress, calcium overload, autophagy, and apoptosis.^6^, ^7^ A series of pathological changes follow infarction, leading to irreversible neuronal damage in the infarct core, which is the main challenge for the treatment of ischemic stroke and a major cause of the poor prognosis.^8^ The hypoperfused area surrounding the infarct core, called the ischemic penumbra, is at risk of delayed cellular injury or even death and may be salvaged by early intervention.^9^

Rabphilin‐3A (Rph3A) is a synaptic vesicle protein from the synaptotagmin‐like superfamily, which contains 684–704 amino acid, has a molecular weight of approximately 75 kDa and varies slightly between species.^10^ Rph3A was first extracted from bovine brain in 1992 and has since been extensively studied as a Rab effector protein involved in presynaptic terminal neurotransmitter release, localized to the membrane of the synaptic vesicles.^11^, ^12^ Later studies showed that Rph3A is densely distributed in synaptic regions of the rat brain, retina, and neuromuscular junction, and dendritic spines of the lateral structural domain of the postsynaptic density (PSD).^13^ Its conformation and activity are regulated by Ca^2+^ ions and IP3; it contains two C_2_ structural domains (C_2_A, C_2_B) near its C‐terminal, which bind Ca^2+^ in a phospholipid‐dependent manner, as well as an N‐terminal Rab3A‐binding structural domain.^13^, ^14^ Rph3A plays an important role in the nervous system. For example, in a transgenic mouse model of Huntington's disease (R6/1), deletion of Rph3A may be responsible for synaptic dysfunction.^15^ In addition, it is involved in processes such as synaptic vesicle transport, neurotransmitter release, stabilization, and retention of NMDA receptors in the postsynaptic membrane, neutrophil polarization, neutrophil–endothelial adhesion, and cell migration. Rph3A is involved in the development of neurodegenerative diseases, such as Huntington's disease, Alzheimer's disease, and pharmacological dyskinesias.^16^, ^17^, ^18^ Importantly, Rph3A is significantly involved in the pathophysiology of reperfusion injury. To the best of our knowledge, no studies have been reported on the role of Rph3A in Cerebral Ischemia and Reperfusion injury (CIRI), and it is unclear whether Rph3A changes during cerebral ischemia‐reperfusion. In the present study, we aimed to explore the association of Rph3A with CIRI, and observe the Rph3A protein levels, mRNA levels and subcellular location to investigate the role of Rph3A in the pathological process of cerebral ischemia‐reperfusion as well as the mechanism underlying it.

## 2 MATERIALS AND METHODS

### 2.1 Animals and ethics

Adult, male Sprague‐Dawley (SD) rats, weighing 280–330 g, were provided by the Animal Centre of the Chinese Academy of Sciences (Shanghai, China). The rats were housed in a quiet place, with relative humidity (40%), constant temperature (23°C) and regular light/dark schedule. The rats had free access to adequate water and food. The animal experimental protocols were approved by the Animal Care and Use Committee of Soochow University and were performed according to the ARRIVE (Animal Research: Reporting In Vivo Experiments) guidelines.^19^

### 2.2 MCAO model establishment

Based on the methods reported previously, we established a middle cerebral artery occlusion (MCAO) model in rats.^20^, ^21^ Briefly, the right middle cerebral artery was occluded for 2 h in SD rats. First, depending on the body weight of the rats, we anesthetized them with an intraperitoneal injection of 4% chloral hydrate (1 ml/100 g).^22^ After anesthesia, the right common carotid artery (CCA), external carotid artery (ECA), and internal carotid artery (ICA) of the rats were exposed using an incision in the middle of the neck. Subsequently, the distal end of the ECA was ligated, proximal end was pre‐ligated (by tying a single wire knot), and an arterial clip was placed distal to the CCA to prevent bleeding during puncture. A bolus wire (0.38 mm in diameter) fused to a hemisphere at the head end and encapsulated with poly‐L‐lysine at the anterior end of the 20‐mm mark was inserted into the right ECA and passed into the right ICA. The bolus wire marker was advanced 1–2 mm past the bifurcation of the ICA and ECA until resistance was met, thereby occluding the right middle cerebral artery (MCA) at the mouth. Afterward, the incision of the ECA was ligated, wound was sutured, and the animal was placed in the rat cage and allowed to awaken. During the procedure, the body temperature was maintained at 36.5–37.5°C. Two hours after molding, the bolus wire was slowly withdrawn under anesthesia, and the animal was returned to the cage for reperfusion (MCAO/R).^23^ The animals were assessed for functional impairment using the Longa grading system to verify accurate occlusion of the MCA after awakening from anesthesia.^24^ Neurological deficit score was graded from 0 to 5. A score of 0 indicated no significant neurological impairment; 1 indicated inability to fully extend the left forelimb; 2 indicated circling to the left when walking; 3 indicated leaning to the left when walking; 4 indicated limb paralysis or inability to walk or loss of consciousness; and 5 indicated death. Animals with a score of 1–4, indicating successful molding, were included in our study. 0 and 5 points are exclusion criteria.

### 2.3 Experiment grouping

#### 2.3.1 Part I: Time‐course analysis of Rph3A protein and mRNA levels after MCAO/R

In *in vivo* experiments, 66 rats (78 rats were included: 66 were operated successfully and survived) were randomly divided into seven groups of six rats each. The experimental groups were divided chronologically into sham, 6, 12, 24, 72, 120 h, and one week after MCAO/R. All rats were executed at the indicated time points after the MCAO/R model was established. RT‐PCR was performed on the ischemic penumbra of six rats each from the sham, 6, 12, 24, 72, 120, and 168‐h groups. Western blot analysis was performed on the penumbra of six rats each from the sham, 6, 12, 24, 72, 120‐h, and 1‐w groups.

#### 2.3.2 Part II: Roles of Rph3A in ischemia‐reperfusion injury in rat brain

In *in vivo* experiments, 228 rats fulfilled the criteria for model establishment (267 used; 228 survived). Of these, 12 rats were randomly divided into two groups (sham group and MCAO/R group) with six rats in each group for immunofluorescence analysis. Additionally, 72 rats were randomly divided into four groups (sham group, MCAO/R group, MCAO/R + Lv‐NC group, and MCAO/R + Lv‐RNAi group) with 18 rats in each group for Western blot validation of downregulated lentiviral effect, TTC staining analysis, and TUNEL staining. We also randomly divided 144 animals into the sham group, MCAO/R group, MCAO/R + Lv‐NC group, and MCAO/R + Lv‐RNAi group, each group had 36 rats, and these 36 rats, 12 rats did rotarod test, 12 rats did adhesive‐removal test, 12 rats did Morris Water Maze test, which are three behavioral experiments.

#### 2.3.3 Part III: Time‐course analysis and subcellular location of Rph3A protein levels after neuronal OGD/R

In *in vitro* experiments, primary cultured neuronal cells were divided into seven groups, namely the control group and OGD‐/R‐treated groups (i.e., 0.5, 1, 3, 6, 12, and 24 h). Cells were collected for Western blot and immunofluorescence staining analyses.

#### 2.3.4 Part IV: Possible mechanisms of effect of Rph3A in ischemia‐reperfusion injury

In this experiment, human‐derived astrocytes were divided into two groups, the control and OGD/R‐treated groups. The cells and medium supernatants were separately collected for immunofluorescence staining and ELISA experiments.

### 2.4 Cell culture

We isolated primary neuronal cells from the brains of 18‐day gestation rat embryos. Efforts were made to minimize the suffering of pregnant rats and embryos as well as the number of embryos used. First, we peeled out the cerebral cortex on both sides of the fetal rat under a microscope and removed the blood vessels and meninges. The brains were digested with trypsin in a 37°C incubator for 6 min. Afterward, the digested brain tissue was blown in a gradient and passed through a sieve while blowing. Then, the filtered fluid was centrifuged for 10 min (1000 rpm/min). The precipitate was collected and resuspended by adding an appropriate quantity of medium to obtain the cell stock solution. After cell counting, neurons were inoculated in 6‐well and 12‐well plates in neural matrix medium (GIBCO, Carlsbad, CA, USA). Neurons were placed in an atmospheric incubator containing 5% CO_2_ at 37°C. The medium was changed every two days for 7–10 days. Finally, neurons were exposed to the indicated treatments, and cells were harvested. Human‐derived astrocytes were cultured in AM (Astrocyte Medium, ScienCell, Cat. No. 1801).

### 2.5 Establishing an *in vitro* oxygen‐glucose deprivation/reoxygenation model

Briefly, the Neurobasal medium was replaced with DMEM (GIBCO) for normally cultured neurons, and the cells were placed in a 37°C atmospheric incubator containing 5% CO_2_ and 95% N_2_ for 2 h. Afterward, the DMEM medium was replaced with the neurobasal medium and transferred to a normal atmospheric incubator for the indicated time period. The control group was incubated in a 37°C atmospheric incubator containing 5% CO_2_ with the neurobasal medium for the same time duration.

### 2.6 Construction of LV‐Rph3A‐RNAi system *in vivo*

Male SD rats, weighing 260–280 g, were anesthetized with an intraperitoneal injection of 4% chloral hydrate (0.1 ml/10 g). Cortical injections of LV‐Rph3A‐RNAi or LV‐NC were administered using a stereotaxic instrument. Injections were administered at three locations in the penumbra of the cortex of each rat as follows: AP+1.0 (site 1), −0.8 (site 2), −2.6 (site 3); ML+3.5; DV −2.5 from the skull. All target sites were ipsilateral to the MCAO. We also injected 3.3 µl of the lentivirus suspension containing 2.5 × 10^8^ TU/ml into each site at a rate of 0.5 µl/min, for a total of 10 µl across the three sites. The microsyringe was left in the place for 5 min after each injection, while the needle was slowly withdrawn and then inserted into the next site. Rats were subjected to the MCAO/R surgery seven days after the lentivirus injection.

### 2.7 TTC staining

The rats were sacrificed with an overdose of the anesthetic, and the brains were quickly removed. The brains were sliced with a razor blade in the anterior to posterior direction to create five coronal slices of 2‐mm thickness each. The brain slices were immersed in a physiological solution containing 2% 2,3,5‐triphenyltetrazolium hydrochloride (TTC) and incubated for 30 min at 37°C in an incubator protected from light.^25^ Infarcted brain tissue was stained white by TTC to contrast with the red‐stained non‐infarcted tissue. The infarcted area was measured using ImageJ software (Rawak Software Inc., Stuttgart, Germany).

### 2.8 RT‐PCR

Total RNA was extracted from the ischemic penumbra of rats using RNA Trizol (Invitrogen, Carlsbad, CA, USA; 15596‐026) according to the manufacturer's protocol. Briefly, 1 ml of TRIzol was added per 50–100 mg of tissue. The tissue was grinded on ice and centrifuged at 12 000 ***g*** and 4°C for 5 min. The precipitate was discarded and left at room temperature for 5 min to allow complete dissociation of the nucleotide–protein complex. After adding 200 µl/ml of chloroform, the solution and shaken vigorously. The solution was left at room temperature for 2–3 min, reacted with chloroform, and centrifuged at 12,000 ***g*** for 15 min. The supernatant was incubated with isopropanol for 5 min and centrifuged at 12,000 ***g*** for 15 min. The pellet was dissolved in RNase‐free water and the concentration of RNA was measured using a spectrophotometer. The RNA was reverse transcribed into complementary DNA (cDNA) using the Revert Aid First Strand cDNA Synthesis Kit (Fermentas, K1622; Thermo Fisher Scientific, Waltham, MA, USA). PCR primers and amplification of GAPDH sequences from the same samples were used as controls (Ribo Biotechnology Co., Ltd., Guangzhou, China). RT‐PCR was performed using DreamTaq Green PCR Master Mix (K1081; Thermo Fisher Scientific). In addition, all PCR reactions were performed using the Gene Amp PCR System 2004 (Perkin Elmer Corp., Norwalk, CT, USA).

### 2.9 Western blot analysis

All of the samples for Western blot analysis were obtained by grinding rat brain tissues or neurons with RIPA lysate (Beyotime, Nanjing, China). Protein concentrations were measured using a specialized assay kit from Beyotime (Beyotime) and the bisquinolinic acid method. Protein samples (20–40 µg/lane) were loaded onto 10% SDS‐polyacrylamide gels, separated, and electrophoretically transferred to NC membranes (Millipore Corp., Milford, MA, USA). The membranes were blocked with 5% bovine serum albumin (BSA, BioSharp, Hefei, China) for 1 h. The membranes were incubated at 4°C with anti‐Rph3A (1:1,000; Santa Cruz Biotechnology, Inc., Santa Cruz, CA, USA) overnight. Primary antibody to β‐Actin (1:3,000; Cell Signaling Technology, Danvers, MA, USA) was used as an internal reference control. The membranes were incubated overnight with the primary antibody at 4°C. The membranes were incubated with horseradish peroxidase‐conjugated secondary antibody (goat anti‐mouse IgG‐HRP, goat anti‐rabbit IgG‐HRP; cell signaling technology) for 1 h at 37°C and washed three times with PBST (PBS + 0.1% Tween 20). Finally, the membranes were displayed using an enhanced chemiluminescence kit (Beyotime). The relative quantities of proteins were determined using the ImageJ program and normalized to the control group. The quantitative analysis was performed by researchers who were blinded to the experimental groups.

### 2.10 Immunofluorescent analysis

For *in vivo* experiments, brain samples were fixed in 4% paraformaldehyde, paraffin‐embedded, and cut into 4‐µm sections. Paraffin sections of brain were dewaxed and treated with citrate antigen repair solution at 100°C, blocked with 1% BSA, permeabilized with 0.1% triton‐100 for 8 min, and then blocked with 5% BSA at 37°C for 1 h. The sections were incubated overnight at 4°C with the primary antibodies and incubated for 1 h at 37°C with the corresponding secondary antibodies. Finally, nuclei were stained with DAPI. All of the experiments after incubation with the secondary antibodies were performed in the dark. For the *in vitro* experiments, the cultured neurons were fixed in 4% paraformaldehyde. The cells were stained with primary and corresponding secondary antibodies, and then the nuclei were stained with DAPI. Finally, paraffin sections and cells were observed under a fluorescent microscope (OLYMPUS BX50/BX‐FLA/DP70; Olympus Co., Tokyo, Japan). At least six random sections of each sample were observed and the representative results are shown. Relative fluorescence intensity was analyzed using the ImageJ program. Quantitative analysis was performed by researchers blinded to the experimental groups. Information on the primary and secondary antibodies used for immunofluorescence was as follows: Rph3A (1: 200; Santa Cruz Biotechnology, Inc.), Neun (1:300), GFAP (1:800), and Iba1 (1:800). Immunofluorescent secondary antibodies were purchased from Life Technologies (Invitrogen) and included the Alexa Fluor‐555 Donkey anti‐rabbit IgG antibody (A21206) and Alexa Fluor‐488 Donkey anti‐mouse IgG antibody (A31570).

### 2.11 Terminal deoxynucleotidyl transferase dUTP nick end labelling (TUNEL) staining

Apoptosis in the brain was detected by TUNEL staining according to the manufacturer's instructions (In Situ Cell Death fluorescein; 11684795910; Sigma‐Aldrich, St. Louis, MO, USA). Briefly, paraffin sections of brain tissue were dewaxed, antigenically repaired, permeabilised in 0.1% Triton X‐100 for 8 min, and washed three times with IF‐PBST. The sections were incubated with the TUNEL staining reagent for 1 h at 37°C (protected from light). Then, the sections were incubated with the primary antibody for NeuN and the corresponding fluorescent secondary antibody. The stained sections were observed under fluorescence microscopy (OLYMPUS BX50/BX‐FLA/DP70; Olympus Corp.). The apoptosis index was defined as the percentage of TUNEL‐positive cells in rat brain tissue. Similarly, the neuronal apoptosis index was defined as the percentage of both TUNEL‐ and NeuN‐positive cells out of the total NeuN‐positive cells in rat brain tissues.

### 2.12 Neurobehavioral evaluation

Each group of 12 rats was tested for behavioral impairment using a scoring system at 24 h after MCAO/R (Longa score; *n* = 6). We tested the motor function, sensory function, and learning and memory abilities of the rats using the rotarod test, the adhesive‐removal test, and the Morris Water maze test, respectively.

#### 2.12.1 Rotarod test

An accelerated rotarod test was used to examine the locomotor capacity of rats before and on days 1, 3, 5, 7, and 14 after MCAO. The diameter of the rotarod spindle was 10 cm. The speed of the spindle was increased from 4 to 30 rpm in 60 s, and 30 rpm was maintained for a maximum of 300 s.^26^ The sensor was triggered and recorded the time when the rats lost their balance and fell from the spindle. The rats were separated from each other by a partition to prevent mutual interference. The apparatus allowed three rats to be tested simultaneously. Prior to testing, each rat was trained three times a day for three days; the last three trials were considered as the baseline. Finally, all rats received an accelerated rotarod test trial on the testing days after MCAO/R.

#### 2.12.2 Adhesive‐removal test

The adhesive‐removal test was performed as reported previously. Briefly, after allowing the rats to become familiar with the test environment, two small pieces of adhesive‐backed paper dots were glued to the wrist of each forelimb. The rats were then returned to the test cage where they typically removed each stimulus by touching it with their teeth. The training was performed five times a day for three days, and the time taken to contact and remove two stimuli from each limb were recorded separately. Rats were included in the experimental group if they were able to contact the dots and remove them within 10 s of the end of the training. Finally, all rats that fulfilled the inclusion criteria were subjected to the specified interventions and completed the MCAO model or sham operation before the adhesive‐removal test was performed.

#### 2.12.3 Morris water maze test

Before the formal test, all rats underwent 3–6 days of Morris water maze training three times a day. Testing was performed on days 22–26 after completion of the indicated interventions and successful MCAO/R modelling or sham surgery to assess neurobehavioral impairment. The arena was 50 cm high and 180 cm in diameter and filled with water to a height of 30 cm, with water temperature of 20–22°C. The platform was placed 2 cm below the water surface. The starting point for each rat varied from day to day. Each test lasted until the rat found the platform and stayed on it for 2 s, or until 60 s had elapsed without finding the platform. After each test, the rats were placed on the platform for 20 s. During each test, the swimming speed, time taken to find the hidden platform, and the length of swimming path to reach the hidden platform were recorded.

#### 2.12.4 Neurological scores

At 24 h after completion of the indicated interventions and successful MCAO/R modelling or sham surgery, neurological tests were performed on six rats from each group to assess the neurological deficits. The scores were assigned according to the previously published Longa 5‐point scale.^27^

### 2.13 Statistical analysis

All data are expressed as mean ± standard error of the mean. GraphPad Prism 8.0 software (GraphPad Prism Inc., San Diego, CA, USA) was used for statistical analysis. Data sets were tested for normality of distribution with the Shapiro–Wilk test. One‐way or two‐way ANOVA, followed by either Dunnett's or Tukey's post hoc test, was used for comparisons with a single or multiple groups, respectively. Statistical significance was set at *p* < 0.05, unless stated otherwise.

## 3 RESULTS

### 3.1 General observations

After the sham surgery or MCAO/R modelling in rats, the body temperature, blood pressure, heart rate, and body weight were dynamically monitored, which did not reveal any significant changes in any of the experimental groups (data not shown). All of the experimental animals were treated as well as possible to reduce suffering. One death occurred in the sham‐operated group, with a mortality rate of 2.7% (2/74 rats), and 37 deaths occurred in the MCAO/R group, with a mortality rate of 13.7% (37/271 rats).

### 3.2 MCAO/R upregulated the levels of Rph3A in rat penumbra neurons and astrocytes

**FIGURE 1:**
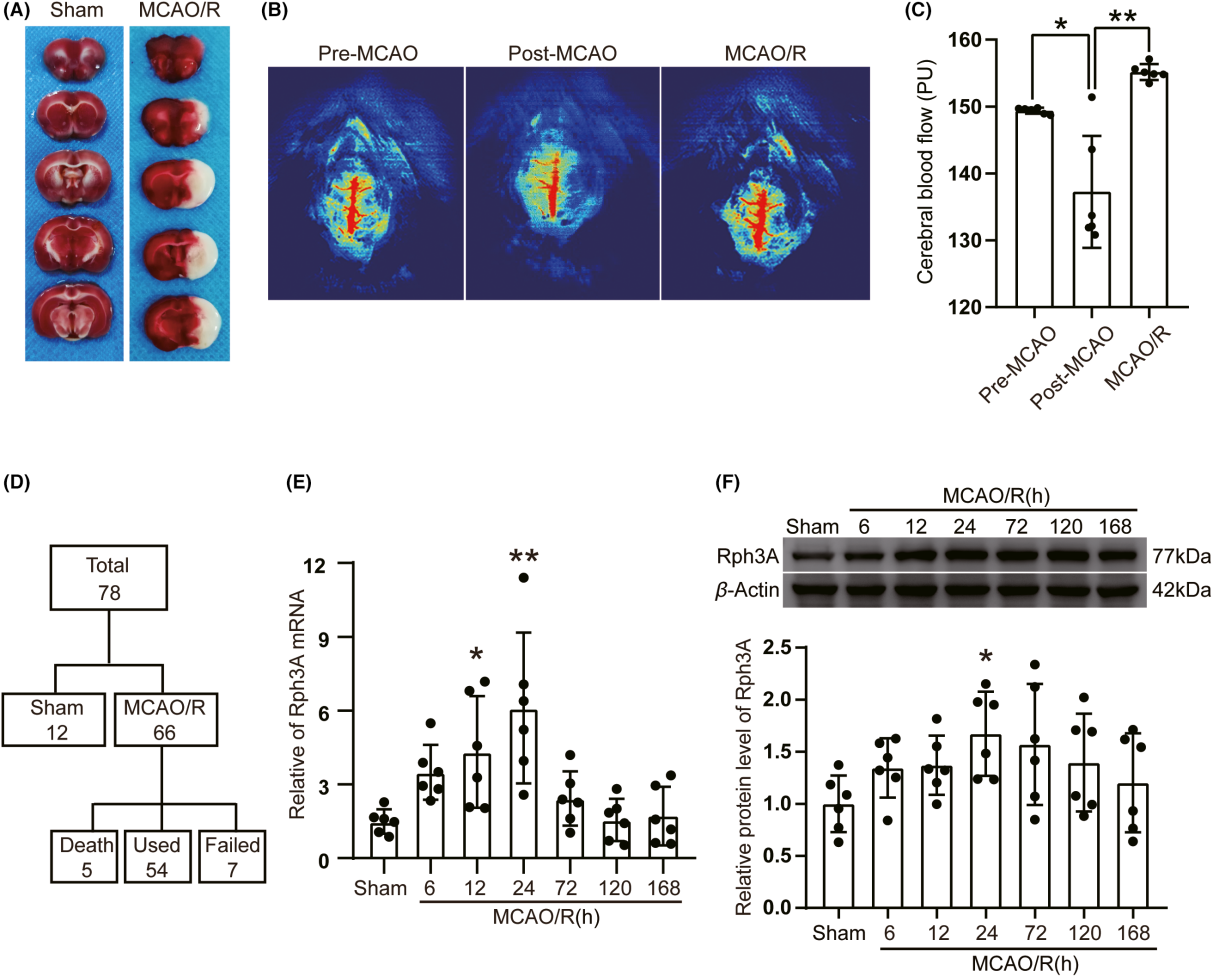
MCAO/R upregulated the levels of Rph3A in rat penumbra neurons and astrocytes. (A) Triphenyl tetrazolium chloride (TTC) staining showing typical MCAO/R 24 h infarction. (B) Laser scatter flow imager showing venous reflux on the surface of the rat brain before, during and after MCAO/R. (C) Cerebral blood flow in rats before and during ischemia and after reperfusion. (D)The success rate of MCAO/R model establishment in this experiment. (E) RT‐PCR quantification of mRNA level of Rph3A in the post‐MCAO/R penumbra and surrounding brain tissue (*n* = 6, *: *p* < 0.05, **: *p* < 0.01). (F) Western blot and quantitative analysis of protein level of Rph3A in the ischemic penumbra and surrounding brain tissue after MCAO/R (*n* = 6, *: *p* < 0.05). Data are presented as mean ± SD. Differences in (C, E and F) were calculated by ordinary one‐way ANOVA

In order to better simulate the pathophysiological process of ischemic perfusion injury in human brain, we established the MCAO/R model in SD rats using transient (2 h) occlusion of the MCA and detected the effect of cerebral infarction in rats by TTC staining and laser scatter flow imaging. There was obvious infarction in the MCA territory of the model group (Figure 1A). The laser scattering flow imager revealed significant differences in venous reflux on the brain surface of the rats before and after modelling and after reperfusion (Figure 1B,C), suggesting that the modelling effect of our MCAO/R model was stable. An analysis of the number of rats modelled in the WB, IF, and RT‐PCR experiments showed a success rate of approximately 82% (Figure 1D). Subsequently, we performed protein blot analysis and RT‐PCR analysis on protein and total RNA samples from the penumbra tissues to verify the changes in protein levels and mRNA levels of Rph3A after MCAO/R, respectively. We found that the mRNA levels of Rph3A in the MCAO/R group gradually increased from 6 h compared to the sham group, with statistically significant differences at 12 h, which increased to higher levels at 24 h, and began to decline at 72 h, falling to a level similar to that of the sham group at approximately 120 h (Figure 1E). Then, we examined the changes in Rph3A protein levels at different time points of brain reperfusion by WB experiments in the ischemic penumbra. Consistent with the RT‐PCR results, the protein levels of Rph3A in the MCAO/R group showed an overall increasing trend followed by a decreasing trend with increasing ischemia‐reperfusion time compared with the sham group; the protein levels increased to the highest level at 24 h and decreased to a level similar to that of the sham group at week 1(Figure 1F).

**FIGURE 2:**
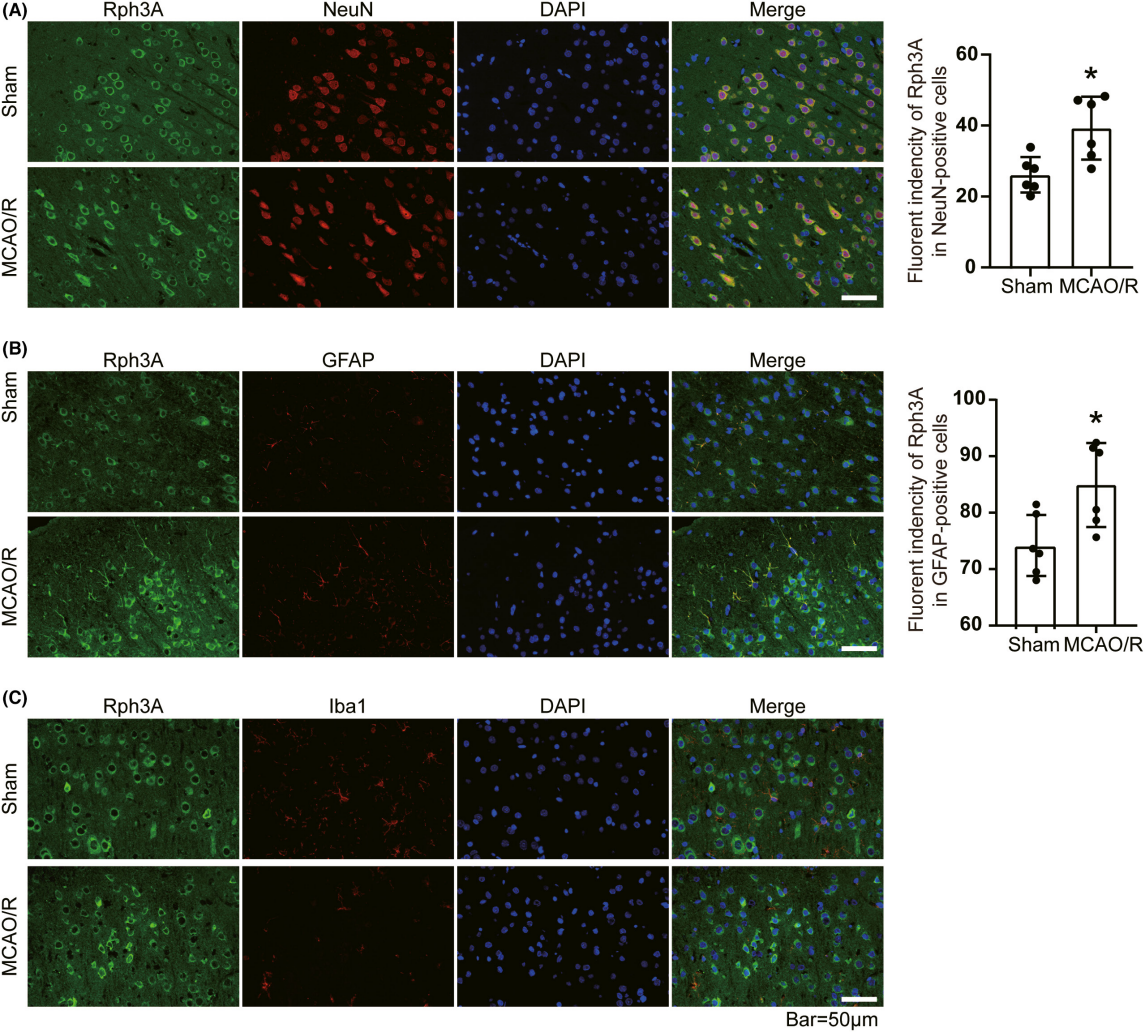
Rph3A protein levels and distribution in different cell types in penumbra of rats. (A) Double immunofluorescence analysis in brain tissue sections with Rph3A antibody (green) and neuronal marker (NeuN, red), nuclei were fluorescently labeled with DAPI (blue), Scale bar = 50 μm. The relative fluorescence intensity of Rph3A in neurons is shown on the right (*n* = 6, *: *p* < 0.05). (B) Double immunofluorescence analysis in brain tissue sections with Rph3A antibody (green) and astrocyte marker (GFAP, red), nuclei were fluorescently labeled with DAPI (blue), Scale bar = 50 μm. The relative fluorescence intensity of Rph3A in astrocytes is shown on the right (*n* = 6, *: *p* < 0.05). (C) Double immunofluorescence analysis in brain tissue sections with Rph3A antibody (green) and microglia marker (Iba1, red), and cell nuclei were fluorescently labeled with DAPI (blue), scale bar = 50 μm (*n* = 6, *: *p* < 0.05)

Previous studies showed that Rph3A is mainly distributed in neurons, anterior, and posterior membranes of synapses and dendritic spines. We examined whether the protein levels and distribution of Rph3A in neurons, astrocytes, and microglia were specific to different cell types using double immunofluorescence in the post‐MCAO (2 h)/R (24 h) penumbra of rats, respectively. The results showed that Rph3A was mainly distributed in neurons and astrocytes (Figure 2A,B), while the protein level of Rph3A was significantly higher in both neurons and astrocytes in the MCAO/R group compared with the sham‐operated group. In contrast, Rph3A was barely detectable in the microglia from the sham and MCAO/R groups (Figure 2C). These results suggest that the distribution of Rph3A in the brain is specific to each cell type.

### 3.3 Downregulation of Rph3A exacerbates cerebral infarction and neuronal death induced by MCAO/R in rats

**FIGURE 3:**
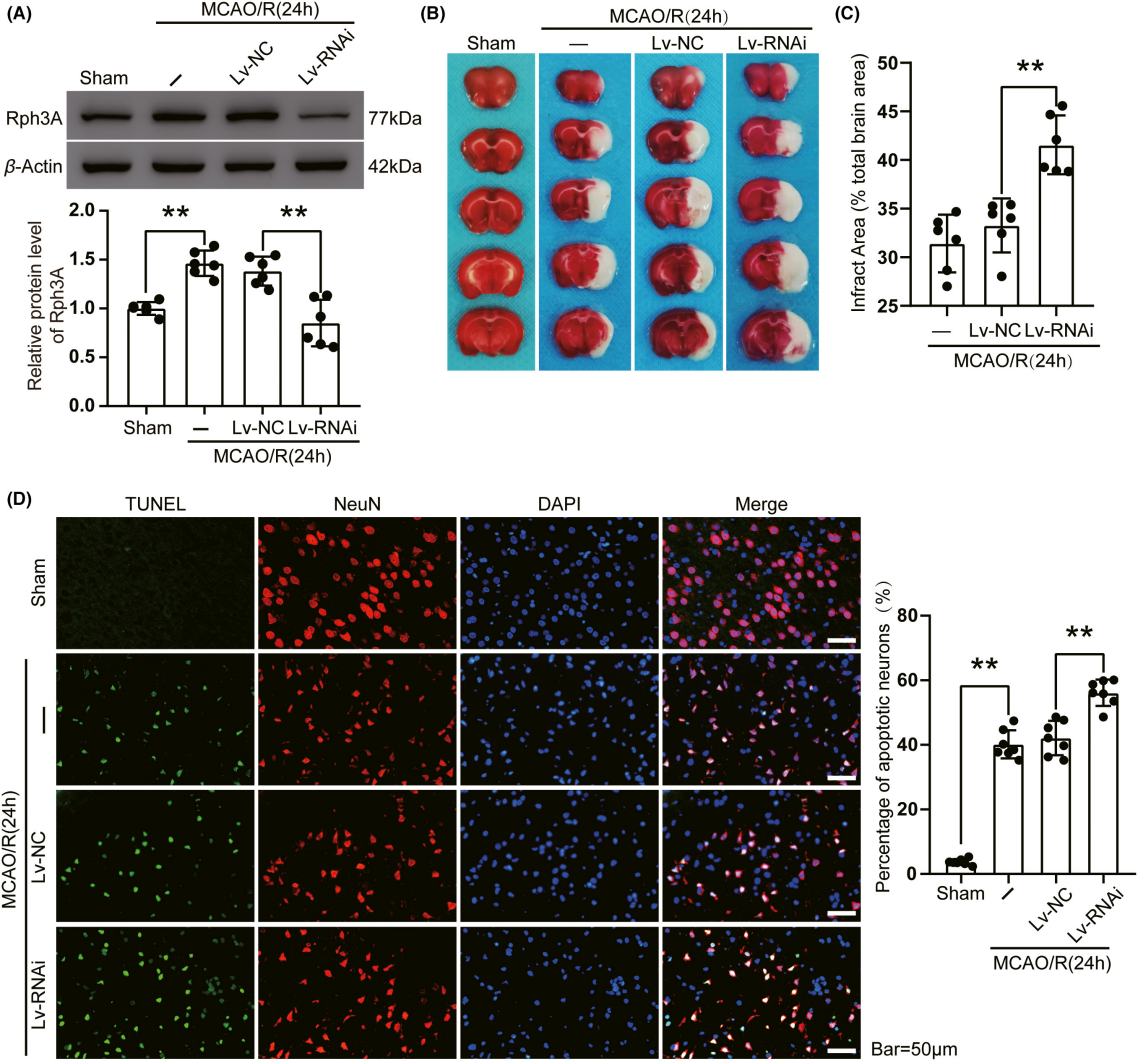
Downregulation of Rph3A exacerbates cerebral infarction and neuronal death induced by MCAO/R in rats. (A) Transfection efficiency of Rph3A lentivirus in rat brain. Quantification of Rph3A protein levels in each group is shown (*n* = 6, LV‐RNAi compared with LV‐NC, **: *p* < 0.01; MCAO/R compared with sham group, **: *p* < 0.01). (B, C) Triphenyl tetrazolium chloride (TTC) staining showed down‐regulation of Rph3A staining of brain sections 24 h after MCAO/R (*n* = 6, **: *p* < 0.01). (D) Double immunofluorescence analysis in brain tissue sections with TUNEL (green) and neuronal markers (NeuN, red), nuclei were fluorescently labeled with DAPI (blue) and apoptotic neurons, that is, both NeuN/TUNEL‐positive cells. Scale bar = 50 μm. The percentage of TUNEL‐positive neurons is shown on the right (*n* = 6, **: *p* < 0.01)

To explore the role of Rph3A in ischemic stroke, we used LV‐RNAi targeting Rph3A to decrease its expression and confirmed the disruptive effect of LV‐Rph3A‐RNAi by Western blotting. The lentivirus significantly reduced MCAO/R‐induced Rph3A protein levels (Figure 3A). Subsequently, we investigated the role of Rph3A in ischemia‐reperfusion‐induced neuronal injury in penumbra. Downregulation of Rph3A resulted in an increased infarct volume in the rat brain, as shown by TTC staining, with an approximately 10% increase in the infarct volume in the MCAO/R + LV‐RNAi group compared with the MCAO/R + LV‐NC group (Figure 3B,C). In addition, double immunofluorescence staining by TUNEL/NeuN showed that TUNEL‐positive neurons were barely observed in the sham group, whereas a significantly higher apoptotic index was found in the MCAO/R group. Additionally, the apoptotic index was higher in the MCAO/R +LV‐RNAi group compared with the MCAO/R +LV‐NC group (Figure 3D). Therefore, downregulation of Rph3A significantly exacerbated neuronal apoptosis.

### 3.4 Downregulation of Rph3A exacerbates neurological dysfunction induced by MCAO/R in rats

**FIGURE 4:**
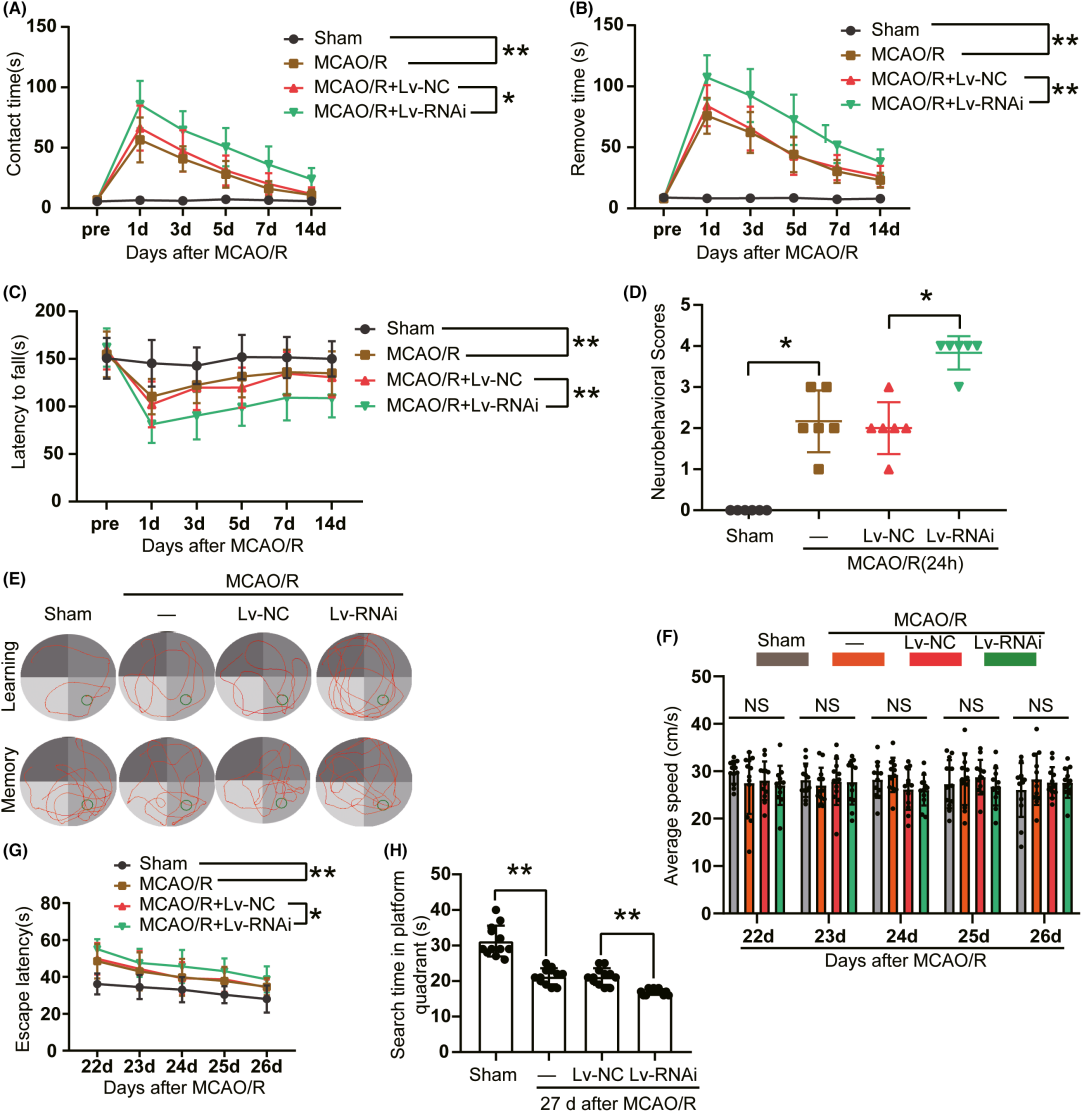
Downregulation of Rph3A exacerbates long‐term neurological dysfunction induced by MCAO/R in rats. (A) The contact time of adhesive‐removal tests in rats after down‐regulation of Rph3A at 1, 3, 5, 7, and 14 days after MCAO/R (*n* = 12, *: *p* < 0.05, **: *p* < 0.01). (B) The removal time of adhesive‐removal tests in rats at 1, 3, 5, 7, and 14 days after MCAO/R (n = 12, **p < 0.01). (C) The latency to fall in rats at 1, 3, 5, 7, and 14 days after MCAO/R in the rotarod test (n = 12, **: *p* < 0.01). (D) Neurobehavioral scores of different groups of rats after MCAO/R (n = 6, *: *p* < 0.05). (E–G) The typical swim path of rats in the Morris water maze test after MCAO/R, representative images illustrate the swim paths at 26 days (learning) and 27 days (memory) after sham surgery or MCAO/R and no significant change was observed in average swimming speed among the four groups (*n* = 12, *: *p* < 0.05, **: *p* < 0.01). (H) The searching time in the quadrant where the original platform was located after downregulation of Rph3A (*n* = 12, *: *p* < 0.05, **: *p* < 0.01)

To verify the effects of downregulation of Rph3A on stroke outcome in rats, we examined the sensory, motor, and learning and memory abilities of rats in the adhesive‐removal test, the rotarod test, and the Morris Water maze test, respectively. The adhesive‐removal test was performed to examine sensorimotor function changes in rats before MCAO and on days 1, 3, 5, 7, and 14 after MCAO/R. On days 1, 3, 5, 7, and 14 after MCAO/R, both the contact time and removal time were shortened in rats, and the shortening was more pronounced in the MCAO/R + LV‐RNAi group compared with the MCAO/R + LV‐NC group (Figure 4A,B). Consistent with this, an accelerated rotarod test was performed to examine the locomotor function of rats before MCAO and on days 1, 3, 5, 7, and 14 after MCAO/R. The results showed that on days 1, 3, 5, 7, and 14 after MCAO/R, the latency to fall in the rotarod test was prolonged in the experimental rats compared with the sham group. Similarly, the latency was significantly prolonged in the MCAO/R + LV‐RNAi group compared with the MCAO/R + LV‐NC group (Figure 4C). At 24 h post‐MCAO/R, we assessed behavioral deficits using the Longa score for each group of six rats. Neurobehavioral test scores were higher in the MCAO/R group compared with sham group and the Longa score was much higher after downregulation of Rph3A compared with MCAO/R group (Figure 4D). Subsequently, the rats were tested for learning ability in the Morris water maze on days 22–26 after MCAO/R. We found that the time to find the hidden platform was significantly longer in the downregulated Rph3A rats compared with the MCAO/R +LV‐NC group, while the sham group had the shortest time (Figure 4E,G). As shown by the results of (Figure 4F), no significant change was observed in average swimming speed among the four groups. Additionally, 27 days after MCAO/R, the platform was removed, and the rats were subjected to the Morris water maze memory test, which revealed that the searching time in the quadrant where the original platform was located was significantly reduced after downregulation of Rph3A (Figure 4E,H). Therefore, downregulation of Rph3A interfered with the spatial learning and memory abilities of the rats after MCAO/R.

### 3.5 OGD/R downregulates Rph3A in cultured neurons

**FIGURE 5:**
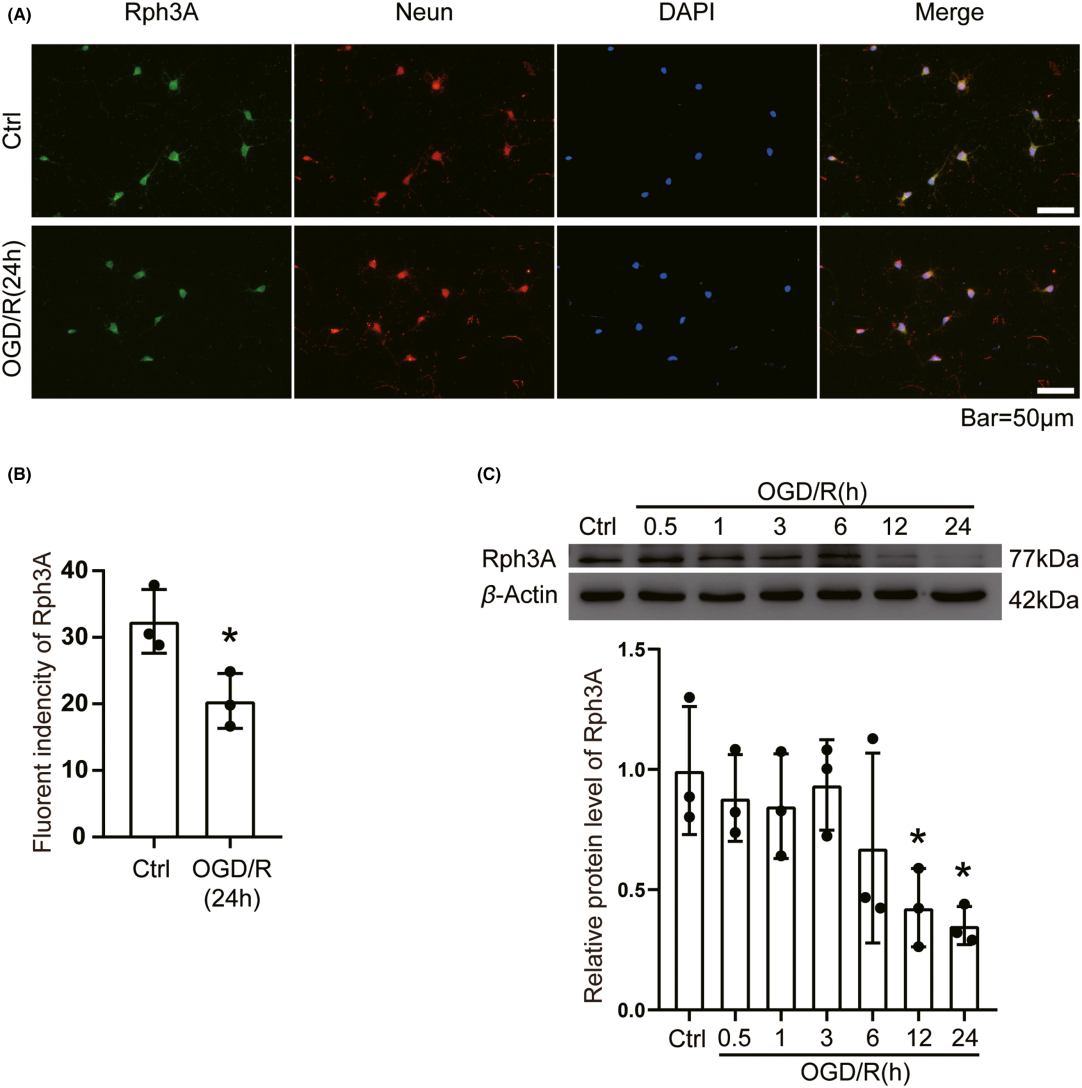
OGD/R downregulates Rph3A in cultured neurons. (A) Double immunofluorescence analysis in cultured neurons with Rph3A antibody (green) and neuronal marker (NeuN, red), and nuclei were fluorescently labelled with DAPI (blue), Scale bar = 50 μm. (B) The relative fluorescence intensity of Rph3A in neurons is shown (*n* = 3, *: *p* < 0.05). (C) Western blot and quantitative analysis of protein level of Rph3A in cultured neurons after OGD/R treatment (*n* = 3, *: *p* < 0.05). Data are presented as mean ± SD. Differences were calculated with Student's *t*‐tests for (B) and ordinary one‐way ANOVA for (C)

To further validate the protein levels and distribution of Rph3A in primary neurons, we used WB and IF methods to detect the changes in protein levels and subcellular localization of Rph3A in primary neurons of rats after oxygen glucose deprivation/re‐oxygenation treatment. We used the double immunofluorescence staining method with anti‐Rph3A and anti‐NeuN and found that Rph3A was distributed within the neuronal cell plasma and on the cell membrane, and that the fluorescence intensity of Rph3A protein decreased by about 55% after neurons were treated with OGD/R (Figure 5A,B). WB results also showed that 2 h after OGD treatment of neurons, the re‐oxygenation time was prolonged and Rph3A protein level gradually decreased. This was demonstrated by a significant decrease in Rph3A protein levels at 12 h compared to the control group, and to even lower levels at 24 h (Figure 5C). These results are in contrast with the *in vivo* studies that found elevated protein levels of Rph3A. Both *in vivo* and *in vitro* experiments had multiple biological and technical replicates for each batch of samples. Therefore, we are confident that the conflicting results between the *in vivo* and *in vitro* are not the result of modelling manipulation methods or experimental techniques. There is extensive intercellular traffic between *in vivo* cells in the brain, such as the neuro‐glial networks. Our *in vivo* findings support the idea that Rph3A plays a neuroprotective role in the ischemia‐reperfusion model. Therefore, we hypothesize that either increased depletion or demand (or both) of Rph3A protein in neurons after MCAO/R exists, and that in the early phase of ischemia‐reperfusion, the body mobilizes Rph3A in glial cells through a regulatory mechanism and delivers it to neuronal cells (e.g., in the form of vesicle secretion) for self‐protection.

### 3.6 OGD/R upregulates the level and secretion of Rph3A in cultured astrocytes

**FIGURE 6:**
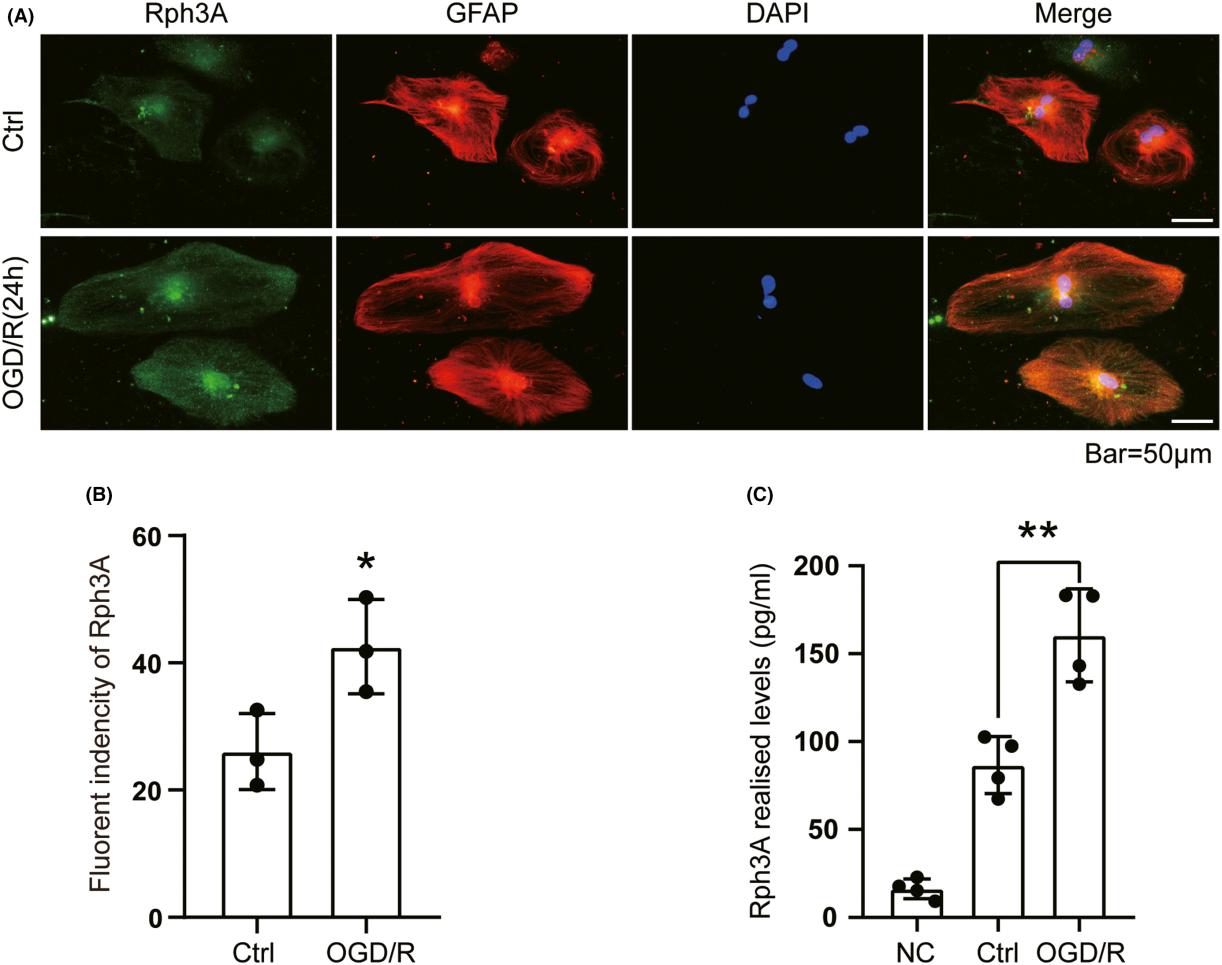
OGD/R upregulates the level and secretion of Rph3A in cultured astrocytes. (A) Double immunofluorescence analysis in cultured human‐derived astrocytes with Rph3A antibody (green) and astrocyte marker (GFAP, red), and nuclei were fluorescently labelled with DAPI (blue), Scale bar = 50 μm. (B) The relative fluorescence intensity of Rph3A in astrocytes is shown (*n* = 3, *: *p* < 0.05). (C) Enzyme‐linked immunosorbent assay (ELISA) was used to detect the protein level of Rph3A in the supernatant of culture medium of human‐derived astrocytes after OGD/R treatment, (*n* = 4, **: *p* < 0.01). NC, negative control; Ctrl, control

Numerous neurobiological studies have demonstrated that glial cells play a major role in most processes in the central nervous system, and astrocytes participate in processes such as energy supply, neurotransmission, and regulation of synaptic excitability. The previous immunofluorescence studies confirmed that Rph3A is distributed in astrocytes; we hypothesized that astrocytes are capable of secreting Rph3A and providing it to neurons to achieve neuroprotection. To test this, we examined the presence of Rph3A in human‐derived astrocytes using double immunofluorescence. Consistent with the results of the immunofluorescence staining of rat brain slices, Rph3A was distributed in human‐derived astrocytes, and the fluorescence intensity of Rph3A after OGD/R treatment was significantly higher than that of the control group (Figure 6A,B). Then, we determined whether astrocytes could secrete Rph3A, so we cultured human‐derived astrocytes *in vitro* and collected the medium after normal culture for 24 h or OGD (2 h)/R (24 h), respectively, and centrifuged the supernatant at 1000 rpm/min for 5 min using a low‐temperature centrifuge. The medium used to culture astrocytes was collected and treated in the same way, and used as a negative control. The change in Rph3A protein concentration in the supernatant of each group was measured using the corresponding ELISA kit. Importantly, Rph3A protein secretion was detected in the supernatant of cultured astrocytes compared with the negative control group, and the concentration of Rph3A in the OGD/R group was higher than that in the normal cultured astrocyte group (Figure 6C). These results suggest that OGD/R treatment promotes increased levels and secretion of Rph3A protein in astrocytes, providing the possibility that astrocytes supply Rph3A to neurons. However, direct evidence and specific mechanisms for the targeted transport of Rph3A from astrocytes to neurons still need to be explored.

## 4 DISCUSSION

Rph3A is a Rab effector protein involved in neurotransmitter release from presynaptic terminals, and its conformation and activity are tightly regulated by Ca^2+^ ions and IP3.^28^, ^29^ Recently, Rph3A has been detected in dendritic spines, where it interacts with GluN2A‐containing NMDARs and promotes their synaptic retention.^13^ Franchini et al. showed that the interaction of Rph3A with GluN2A is required for the stabilization of NMDARs at PSD synapses in posterior hippocampal neurons induced by long‐term potentiation (LTP) and for triggering LTP synaptic adaptation and downstream signaling of cognitive behavior.^16^, ^30^, ^31^ Although Rph3A has been studied for decades, studies have not directly measured the changes in protein levels and mRNA levels of Rph3A induced by cerebral ischemia‐reperfusion. Whether it is equally important in cerebral ischemia‐reperfusion, and its exact mechanism remain uncertain. Herein, we point to a protective role of Rph3A against ischemia‐reperfusion injury. We explored the role and possible mechanisms of Rph3A in reperfusion by means of *in vitro* and *in vivo* ischemic stroke models. *In vivo*, downregulation of Rph3A levels by Lv‐Rph3A‐RNAi affected the sensory function, motor function, spatial learning, and memory deficits in rats, exacerbated apoptosis of neurons in the penumbra, and expanded infarct size, thereby confirming this finding.

Our cellular proteomics data suggest that Rph3A protein levels are decreased in OGD/R‐treated neuronal cells. We found that in primary neuronal cells cultured in vitro, Rph3A was predominantly distributed in the cell plasma and cell membrane, and that Rph3A decreased by approximately 50–60% after OGD/R. In contrast, *in vivo* WB and IF experiments revealed that Rph3A was expressed in both neuronal cells and astrocytes, and that protein levels of Rph3A were significantly increased after MCAO/R. Importantly, we observed that this result was inconsistent with the results of the *in vivo* experiments. Based on the fact that Rph3A plays an important role in vesicle transport, we speculate that in the early stages of cerebral ischemia‐reperfusion, the organism mobilizes Rph3A in astrocytes and delivers them to neuronal cells in a specific manner (e.g., in the form of vesicular secretion) for self‐protection through regulatory mechanisms in response to the sudden injury. The results of the next ELISA experiments support our idea that astrocytes are able to secrete Rph3A, and that Rph3A secretion is significantly increased after OGD/R treatment. In addition, further *in vivo* experiments revealed that downregulation of Rph3A by Lv‐RNAi not only aggravated the apoptosis of penumbra neurons and increased the area of infarction but also impaired the sensory function, motor function and spatial learning and memory abilities of the rats. Our results suggest that Rph3A plays a neuroprotective role in ischemia‐reperfusion injury, but the exact mechanism remains unclear. It has been shown that Rph3A expression at striatal synapses and its interaction with GluN2A‐containing NMDAR are increased in a rat model of Parkinson's disease.^32^ GluN2A‐containing NMDARs translocate to the synapses due to the mobilization of pre‐assembled NMDARs from non‐synaptic pools.^33^ However, the selective aggregation of these receptors is because Rph3A selectively binds to GluN2A, but not to other GluN2‐type regulatory NMDAR subunits. Rph3A interacts with GluN2A/PSD‐95 to form a ternary complex, thereby affecting the stability and retention of NMDARs at the synapse. Notably, in this scenario, Rph3A is not associated with newly synthesized proteins.^16^ Our findings revealed that Rph3A protein levels decreased in an *in vitro* model of ischemia‐reperfusion and increased *in vivo* after MCAO/R. This increase may not be due to increased synthesis, but rather increased translocation. Furthermore, in studies of cellular models of Alzheimer's disease, it was found that in hippocampal cells, GluN2A and GluN2B of astrocytes were able to counteract the synaptotoxic effects of anti‐amyloid beta (Aβ) and that this synaptic protection may be achieved by paracrine secretion of β‐neurotrophic factor.^34^, ^35^ However, Aβ treatment upregulated levels of GluN2A and GluN2B in hippocampal astrocytes from mixed neuron‐astrocyte cultures, but not in pure astrocyte cultures.^34^ We assumed the existence of a neuronal cell‐glial cell network *in vivo*, where Rph3A protein levels decreased after OGD/R treatment in cultured primary neuronal cells. However, *in vitro*, neuro‐glial cell communication was artificially severed and Rph3A levels could not be replenished; whereas *in vivo*, the elevated Rph3A protein levels in neuronal cells after MCAO/R may be because in the early phase of ischemia‐reperfusion, the body mobilizes astrocytes and delivers Rph3A to neuronal cells for self‐protection in response to the sudden injury. Rph3A may play an important role in the neuronal‐glial cell network. The most important point, of course, is that in the future studies we need to explore the mechanisms underlying the secretion and targeting of Rph3A for transport to neuronal cells.

Rph3A is also involved in vesicle transport, exocytosis, and release of neurotransmitters. The release of presynaptic neurotransmitters is tightly regulated by SNARE proteins, Ca^2+^ and many Ca^2+^‐sensitive proteins, including synaptic binding proteins (Syts) and double C_2_ domain proteins (Doc2s).^36^ The concept of spontaneous release was introduced more than 70 years ago, but the mechanisms regulating this process are still poorly understood. Syt‐1, Syt7, and Doc2 proteins are mainly, but not exclusively, involved in synchronous, asynchronous, and spontaneous release phases. They share the same conserved tandem C_2_ domain structure but have different functions in terms of subcellular location, Ca^2+^ binding properties and protein interactions.^37^ In a study of spontaneous release from hippocampal glutamatergic neurons, it was found that ablation of the Rph3A gene in the absence of Doc2 and removal of Rph3A resulted in an increased frequency of spontaneous release from glutamatergic neurons in neuron‐glia co‐cultures, suggesting that Rph3A may influence spontaneous release of glutamate from hippocampal neurons and play a regulatory role in the neuronal network.^38^ An earlier study on the correlation between Rph3A and neurotransmitter release was carried out by Burns et al.^28^, who found that presynaptic neurotransmitter release was inhibited after injection of recombinant full‐length bovine Rph3A into the giant presynaptic terminals of squid. However, the exact mechanism by which Rph3A inhibits neurotransmitter release was not known until it was revealed that in PC12 cells, Rph3A can interact with synaptosomal‐associated protein (SNAP25) during the cytosolic docking step.^39^, ^40^ Previous studies in our laboratory found that ANXA7 can interact with SNAP25 and promote SNAP25‐mediated presynaptic glutamate release, which is involved in the pathological process of secondary brain injury after cerebral hemorrhage in rats.^41^ Excitatory amino acid toxicity plays an important role in reperfusion injury. Since Rph3A inhibits neurotransmitter release and interacts with SNAP25, it is possible that their complex could affect presynaptic glutamate release. If the Rph3A/SNAP25 complex can inhibit presynaptic glutamate release, this may be a mechanism by which Rph3A exerts its neuroprotective effects. Therefore, future studies should explore whether Rph3A and SNAP25 can interact and inhibit the release of presynaptic glutamate in the MCAO/R model.

Our study has several limitations. Our current findings can only speculate that neurons can take up Rph3A released by astrocytes, and there is no direct evidence. Therefore, we will use the AAV characteristic of astrocytes to track Rph3A in our following study. Additionally, although our study showed that *in vivo* Rph3A has a protective effect on neurons, whether it has the neuroprotection *in vitro* needs further validation. Finally, only male rats were tested in the current study. However, sex differences exist in cerebral vessels, metabolism, ischemic neuronal injury, and stroke outcomes.^42^, ^43^, ^44^, ^45^, ^46^ Lack of data on both sexes is considered a limitation of this study, which should be discussed with future plans.

## 5 CONCLUSIONS

The present study showed that ischemic stroke induces an increase in Rph3A protein levels and mRNA levels in neuronal cells, and the quantity of astrocytes in the penumbra, and that the increase in protein levels may be associated with increased secretion and targeted transport to neuronal cells by astrocytes. In addition, to the best of our knowledge, the distribution of Rph3A in astrocytes has not been reported previously. Finally, increased levels of Rph3A protein have a protective effect on neurons in the penumbra. This finding provides a new direction for studies on the rescue of penumbra after ischemic stroke.

## CONFLICT OF INTEREST

The authors declare that they have no competing interests.

## AUTHOR CONTRIBUTIONS

Xianlong Zhu and Haiying Li contributed to the conception of the study. Wanchun You and Zongqi Wang performed the data analyses and wrote the manuscript. Hao Yu and Zhong Wang contributed significantly to analysis and manuscript preparation. Haitao Shen and Xiang Li helped perform the analysis with constructive discussions. Zhengquan Yu and Gang Chen played an important role in interpreting the results.

## Data Availability

The data that support the findings of this study are available from the corresponding author upon reasonable request.
